# Mental Schemas Hamper Memory Storage of Goal-Irrelevant Information

**DOI:** 10.3389/fnhum.2015.00629

**Published:** 2015-11-26

**Authors:** C. C. G. Sweegers, G. A. Coleman, E. A. M. van Poppel, R. Cox, L. M. Talamini

**Affiliations:** ^1^Department of Psychology, University of AmsterdamAmsterdam, Netherlands; ^2^Department of Psychiatry, Beth Israel Deaconess Medical CenterBoston, MA, USA; ^3^Department of Psychiatry, Harvard Medical SchoolBoston, MA, USA

**Keywords:** episodic memory, schemas, EEG, congruency, old/new effect

## Abstract

Mental schemas exert top-down control on information processing, for instance by facilitating the storage of schema-related information. However, given capacity-limits and competition in neural network processing, schemas may additionally exert their effects by suppressing information with low momentary relevance. In particular, when existing schemas suffice to guide goal-directed behavior, this may actually reduce encoding of the redundant sensory input, in favor of gaining efficiency in task performance. The present experiment set out to test this schema-induced shallow encoding hypothesis. Our approach involved a memory task in which faces had to be coupled to homes. For half of the faces the responses could be guided by a pre-learned schema, for the other half of the faces such a schema was not available. Memory storage was compared between schema-congruent and schema-incongruent items. To characterize putative schema effects, memory was assessed both with regard to visual details and contextual aspects of each item. The depth of encoding was also assessed through an objective neural measure: the parietal old/new ERP effect. This ERP effect, observed between 500–800 ms post-stimulus onset, is thought to reflect the extent of recollection: the retrieval of a vivid memory, including various contextual details from the learning episode. We found that schema-congruency induced substantial impairments in item memory and even larger ones in context memory. Furthermore, the parietal old/new ERP effect indicated higher recollection for the schema-incongruent than the schema-congruent memories. The combined findings indicate that, when goals can be achieved using existing schemas, this can hinder the in-depth processing of novel input, impairing the formation of perceptually detailed and contextually rich memory traces. Taking into account both current and previous findings, we suggest that schemas can both positively and negatively bias the processing of sensory input. An important determinant in this matter is likely related to momentary goals, such that mental schemas facilitate memory processing of goal-relevant input, but suppress processing of goal-irrelevant information.

**Highlights:**

– Schema-congruent information suffers from shallow encoding.
– Schema congruency induces poor item and context memory.
– The parietal old/new effect is less pronounced for schema-congruent items.
– Schemas exert different influences on memory formation depending on current goals.

## Introduction

A hierarchical organization with feedforward and feedback connectivity between hierarchical layers is pervasive throughout the nervous system (Felleman and Van Essen, 1991; Rockland et al., 1997). In this organization, interactions between feedforward and feedback activity determine what is represented at any given level. The feedback activity in the network is thought to bias competitive processing in line with prior information, thus biasing attention, as well as inferences about the sensory input (Felleman and Van Essen, 1991; Rockland et al., 1997; Markov and Kennedy, 2013). At the highest level of the processing hierarchy such prior information consists of schemas: generalized, higher-level constructs that encompass configurational (associative) similarities across events, rather than the specificity that makes those events unique (for other definitions, see Fiske and Linville, 1980; Ghosh and Gilboa, 2013 or van Kesteren et al., 2013). It is thought that such schemas are extracted across experiences through a hippocampo-neocortical dialogue (Sweegers et al., 2014). A number of recent studies have addressed the influence of such schemas, plausibly stored at the level of distributed neocortical networks, on the encoding and consolidation of hippocampus-dependent, episodic memories (Tse et al., 2007, 2011; van Kesteren et al., 2010, 2013; Sweegers et al., 2014). Overall, these studies find that schemas aid the storage of schema-congruent information. This means that memory for items that are in line with, or that can be related to a schema are better memorized than items that are incongruent to the schema. One of the mechanisms underlying this memory benefit may regard the acceleration of hippocampus-dependent consolidation mechanisms (Tse et al., 2007; McClelland, 2013).

In the experiments thus far, memorizing the so-called “schema-congruent” items was typically rewarding. That is, memorizing the specifics of the individual items served the pursuit of an internal goal. For example, in a rat study, extra food was delivered when these items were retrieved (Tse et al., 2007), and motivation was boosted in a human study through announcement of an upcoming memory test (van Kesteren et al., 2013). It has not been investigated how memory formation proceeds when the storage of new schema-congruent input is redundant or irrelevant, given the goal at hand; that is, when behavior in pursuit of the goal can fully depend on existing schemas, without an important benefit from storing individual schema-congruent items. Given competitive principles in neural processing, mental schemas may then lead to shallow encoding of goal-irrelevant information, thus increasing behavioral speed and preserving processing capacity for more relevant computations.

Many studies support the notion that shallow encoding leads to qualitatively poor memory traces, holding few visual details and contextual aspects from the learning period. In such studies encoding strength has been manipulated through, for instance, stimulus presentation time (Vilberg and Rugg, 2009), encoding task (Craik and Tulving, 1975), attention at encoding (Craik et al., 1996; Naveh-Benjamin et al., 2003) or the number of stimulus repetitions (Nelson, 1977; Rugg and Doyle, 1994). The influence of such encoding manipulations on memory is also reflected in the electroencephalography (EEG) during memory retrieval. In particular, in the parietal old/new effect: an event related potential (ERP) effect, occurring between 500 and 800 ms after stimulus onset over parietal areas of the scalp (Allan et al., 1998; Friedman and Johnson, 2000; Yonelinas, 2002). This ERP effect reflects higher EEG amplitudes evoked by correctly recognized old items than by correctly rejected new items (Parker et al., 2003). Importantly, the aforementioned studies on encoding strength manipulations have shown that only vivid memories, containing rich contextual detail from the learning period (created under deep encoding conditions), elicit this effect, whereas memories that hold little contextual detail (created under shallow encoding conditions) do not. In other words: the presence of this ERP is thought to reflect recollection: the ability to retrieve source information from the study episode (Wilding, 2000; see Yonelinas, 2002 for a review on recollection processes). If schemas induce shallow encoding, visual detail and context memory will suffer and the parietal ERP will be diminished.

A previous study in our lab (Sweegers and Talamini, 2014) that investigated the formation of schemas from episodic memory provided some first support for *schema-induced shallow encoding*. We found that the extraction of associative regularities from the learning material (associative exemplars) negatively influenced the formation of detailed memory traces for the individual exemplars, reducing the incorporation of regularity-irrelevant details. In the present experiment we build upon this finding by having subjects now learn a regularity structure, or schema, before encoding. The aim is to study the influence of existing mental schemas on the encoding of new information.

To address this issue, we had subjects perform a task in which faces had to be associated to a limited set of homes (a house, a caravan, a tent, etc.). Before encoding, subjects learned a schema consisting of several rules regarding the relation between certain combinations of facial features and particular homes (for example: stout faces with no headwear go with the caravan). During encoding, a schema of this sort predicted the corresponding home for half of the faces, whereas the other half was randomly assigned to non-schema homes. Subjects learned to pick the corresponding home for each face. The rationale was that the schemas would hamper the formation of a detailed memory representation for the congruent items, as there was, in theory, no need to create memory representations of these items to perform the task at hand. Shortly after encoding we assessed the storage of visual detail by testing face recognition against highly similar lures and new faces, under EEG recording. We expected recognition performance to be inferior for schema-congruent faces as compared to schema-incongruent ones. For the EEG analysis, we focused on the parietal old/new effect. We hypothesized that this ERP effect would be less pronounced in the schema-congruent condition than the schema-incongruent condition, reflecting the retrieval of contextually poor memories for schema-congruent items. Finally, we tested context memory behaviorally, as indexed by memory for the location of the home that was coupled to each face during encoding. Also here, we expected performance to be lower for schema-congruent items than schema-incongruent items.

## Materials and Methods

### Subjects

Fifty-four subjects gave written informed consent and received either course credits or financial compensation for participation in this experiment, which was approved by the local ethics committee. Six subjects were excluded from the experiment as they did not reach the pre-set number of trials during the practice session (see below). Another twelve were excluded from the EEG analyses due to technical problems (3), having fewer than 10 artifact-free trials per condition (3) or not showing the schema manipulation in their behavioral responses (6, see below). Behavioral analyses were thus performed on 48 subjects (14 males, mean age 20.60, *SD* = 1.62); EEG analyses on 36 subjects (11 males, mean age 20.58, *SD* = 1.61).

### Overview

The following gives an overview of the experimental design; please see the sections below for methodological details. The experiment was spread across two consecutive days. On day 1, subjects were familiarized with the face-home task and asked to memorize several rules regarding combinations of facial features and homes, which together made up the schema. On day 2, allowing for some consolidation of the schema, subjects took part in the learning session. This entailed learning 72 associations of faces with homes. Half of the associations were congruent with the schema; the other half was random. After the learning session, subjects were prepared for EEG recording. In the ensuing test phase, recognition memory for both the schema-congruent and the schema-incongruent faces was assessed under EEG recording. Finally, context memory was assessed by asking subjects to pick the location of the home that belonged to the face in the learning phase.

### Stimuli

**Figure 1:**
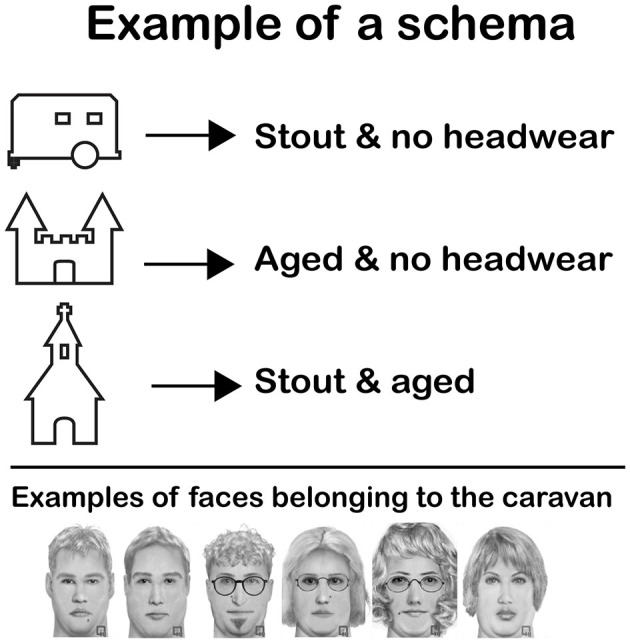
**Example of a schema.** Subjects were asked to memorize a schema such as this one before the start of the practice session on day 1. The schema taught them that a certain type of faces was always connected to the same home. In this particular example, “stout and no headwear” faces were always coupled to the caravan. The castle and the church also had a certain type of faces coupled to them. The figure shows six faces that could be coupled to the caravan (all stout and no headwear). Note that although all face examples are from the same face category, they are perceptually quite different.

Grayscale pictures of emotionally neutral faces were created using Faces TM software (Biometrix, 2003). The faces varied on several non-critical features. However, three systematically manipulated, binary features occurred in each face: faces were either: (1) young adult or aged; (2) slender or stout and had either; (3) headwear (caps, hats or headbands) or no headwear. These critical facial features could come in various forms (e.g., different types of headwear, wrinkle patterns, etc.), contributing to the perceptual distinctiveness of the faces (see Figure 1). Six out of the eight possible three-way combinations of these features were selected for use in the experiment. Each of these six face categories was thus characterized by a unique combination of three critical features. However, for each face category just 2 (out of the 3) critical features sufficed to distinguish that category; this 2-feature combination did not occur in any other category (this circumstance is intrinsic to the systematic counterbalancing of critical features during item construction). For each of these six face categories 24 faces were created. Twelve faces from each category (72 in total) were used in the learning phase. With photo-editing software (Adobe Photoshop), these 72 faces were slightly modified to create 72 additional lure faces (changes mainly involved the size and shape of the eyes, nose and mouth). In the face recognition test we presented subjects with 216 faces: the 72 faces from the learning phase, the 72 similar lures and the remaining 72 original faces (appointed as “new faces”). Finally, 24 extra faces (four from each category) were created with Faces TM software to serve as practice faces for day 1, and another 10 (from various categories) served as examples.

Line drawings (six in total) of a house, church, factory, tent, castle and caravan were created such that face-home associations could be made. Three of these were appointed as “schema-congruent homes”, meaning that all 12 faces associated with that home belonged to the same face category. The other three homes were “schema-incongruent homes” and the faces in the three remaining face categories were randomly assigned to these homes. Thus, only for three homes, the associated faces could be predicted from the schema: the unique combination of two facial features that occurred in a particular face category fully determined with which home it was paired.

The assignment of homes and face categories to either the schema-congruent or schema-incongruent condition was semi-randomized across subjects.

### Day 1: Schema Learning and Practice

**Figure 2:**
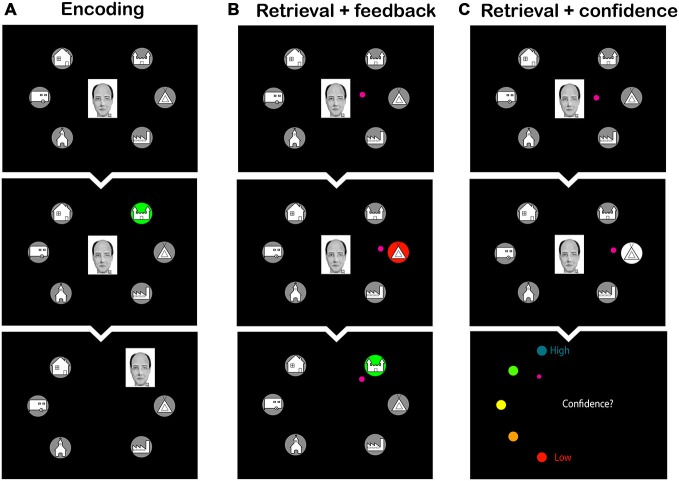
**Screen shots from the learning phase of the face-to-home association task. (A)** Encoding phase: subjects observed the faces moving to their corresponding home. **(B)** Retrieval phase with feedback: subjects were asked to select the home that was associated with the face. Feedback was provided by presenting the wrong home in red, and subsequently the correct home in green. **(C)** Retrieval phase with confidence rating: subjects were asked to select the home corresponding to the face and to make a confidence judgment thereafter. The small pink circle is the cursor that the subjects had to move to make their choice.

After arriving at the lab, subjects received a sheet of paper with the schema and were asked to memorize it. They were informed that the schema would greatly aid in connecting the faces to the homes in an upcoming memory task and that they should use the schema whenever possible. The practice phase followed, in which subjects had to associate 24 faces, 12 schema-congruent and 12 schema-incongruent ones, with their corresponding homes in two encoding-retrieval cycles. During an encoding block, each of the 24 faces was presented for 1 s over a mid-screen fixation cross, then moved to one of the six homes that were organized hexagonally around the fixation cross, and stayed there for another 1.5 s (see Figure 2A). Immediately after each encoding block, a retrieval block followed, in which subjects were instructed to indicate the correct home for each face. Faces were presented sequentially, for 2 s each, over the fixation cross. Subjects used a joystick to move the cursor from the fixation cross to the selected home and confirmed their choice with a button press. In the first retrieval block, subjects received feedback on each placement: if the correct home was chosen, the home turned green, and the face moved to that location; if an incorrect location was chosen, the home turned red, and the correct home turned green (see Figure 2B). Subsequently, the subject had to make a movement to the correct home, after which the face moved to that home. In the second retrieval block, no feedback was given, but subjects had to indicate their response confidence on a five-point scale (1 = low to 5 = high; see Figure 2C). The order of the faces was randomized over blocks and over subjects.

Importantly, there was no systematic relation between homes and screen locations. However, for a particular face-home association the home always appeared on the same location. Hence, the location of the home can be seen as a contextual aspect to the more central face-home association that could be implicitly learned during the task.

At the end of the practice session, subjects needed to have a good understanding of the task layout. Subjects who did not pick the correct homes for at least 9 out of 12 schema-congruent faces and 6 out of 12 incongruent faces were excluded from the experiment. We reasoned that subjects who did not meet this criterion did not have a good understanding of the task, which required switching between schema and non-schema strategies in order to obtain maximum performance.

### Day 2: Learning and Test Phase

Upon arrival at the lab subjects were instructed to learn 72 new face-home associations. They were asked to use, where possible, the previously learned schema for connecting the 72 new faces with their corresponding homes. Three encoding-retrieval cycles followed; the first two included feedback, while the third included confidence rating instead of feedback. Apart from the stimuli and the number of encoding-retrieval cycles, the encoding procedure was exactly the same as on day 1 (*duration* = 45 min). After a 10 min break, subjects were prepared for EEG recording, which took approximately 1 h. Thereafter they performed a 30 min surprise face recognition test, under EEG recording.

During face recognition, the 72 faces from the learning phase, 72 lure faces and 72 new faces were presented, intermingled. The lure faces were introduced to assess whether subjects’ face memories incorporated sufficient detail to distinguish between previously seen faces and faces that were very similar. The new faces were included for the parietal old/new ERP investigation.

For this task, faces were presented sequentially, in the middle of the screen, for 750 ms each. Each face was followed by the question “Old, lure or new?” which appeared for 3 s. Subjects were instructed to use buttons on the joystick to answer the question within the 3 s interval. If subjects did not respond within the allotted time interval an omission was scored. After those 3 s, subjects made a confidence judgment (self-paced).

After a short break, subjects performed the contextual memory task, which required them to pick the location of the home that corresponded to each face. As mentioned briefly before, the location of the home can be seen as a contextual aspect to the more central face-home association that could be implicitly learned during the task. Each of the 72 learned faces was shown for 2 s in the middle of the screen, which also showed a hexagonal pattern of six gray circles, reflecting the positions of the homes during learning. Subjects responded by selecting one of the gray circles with the cursor, after which confidence was rated. This contextual memory task was self-paced and lasted approximately 10 min.

Finally, a questionnaire was administered to assess whether subjects intentionally encoded the locations of the homes along with the face-home associations during encoding. In the current experiment, the location is intended as contextual to the more central face-home association. Subjects’ intentional use of location information for task performance would confound the interpretation of the contextual memory results.

### EEG Acquisition and Analyses

EEG was recorded using a 64-electrode ANT Waveguard EEG cap with, in addition, two mastoid electrodes as reference and four electrodes for horizontal and vertical electro-oculography (72-channel Refa DC amplifier (TMS International, Enschede, Netherlands), sampling rate: 512 Hz, impedance below 20 kΩ). EEG data was analyzed using the EEGLAB toolbox in Matlab (Delorme and Makeig, 2004). Break periods were manually removed from the continuous filtered EEG (high pass filter: 0.1 Hz, notch filter: 50 Hz) and bad channels were interpolated. Independent Component Analysis (ICA) was used to remove eye blinks, eye movements, and other noise components from the continuous EEG data (noise rejection was based on criteria published by the NBT: www.nbtwiki.net/lib/exe/fetch.php?media=tutorial:artifacts_2012.pdf). Next, EEG data from the face recognition test was epoched from 200 ms before stimulus onset (for the baseline correction) to 2 s post-onset and epochs containing excessive artifacts (>75 μV) were removed.

For the investigation of the parietal old/new effect we included old and new items (so no lure items). Importantly, for the old items, we included only those trials in which subjects gave an “old” or “lure” response, so as to incorporate all recognition responses, including both detailed and more approximate ones, in the ERP analyses. For the new items, we included only those trials in which subjects gave a “new” response (the correct rejections). This resulted in four conditions of interest: schema-congruent old faces, schema-congruent new faces, schema-incongruent old faces and schema-incongruent new faces. Please note that lure faces were not used for the EEG analyses, but were included for behavioral analyses only.

The parietal old/new effect was analyzed considering two regions of interest (ROIs): a left parietal cluster (including P1, P3, P5, PO3 and CP3) and a right parietal cluster (P2, P4, P6, P04, CP4; see Yu and Rugg, 2010; Mollison and Curran, 2012; Wolk et al., 2013 for similar cluster-based analyses). Per subject, EEG data was first averaged across trials in a condition, and then averaged across the five electrodes in a ROI. Finally, a single mean amplitude was calculated, by averaging across the time points within our time window of interest (500–800 ms; Woodruff et al., 2006; Speer and Curran, 2007; Yu and Rugg, 2010; Mollison and Curran, 2012). These averaged EEG amplitudes per subject, per condition, per ROI were used for group-level analyses.

As we were interested in studying the neural correlates of a potential schema effect, data from subjects that did not show the schema manipulation in their behavioral responses was excluded, in order to improve the signal-to-noise ratio. To quantify the schema effect on an individual basis, we first summed the correct identification scores of old and lure faces for each schema condition separately. Next, the summed score in the schema-congruent condition was subtracted from the summed score in the schema-incongruent condition. Thirty-six subjects had positive difference scores whereas a mere six showed either no difference or a negative difference score. These six subjects were excluded from the EEG analysis.

## Results

### Behavioral Results

As the behavioral data was largely non-normally distributed, Wilcoxon Paired Signed Rank tests were used to compare the schema-congruent and schema-incongruent conditions. As there were 36 faces in each condition, this was always the maximum score.

### Face to Home Association

During the learning phase, subjects could make use of the schema to connect the schema-congruent faces to their homes. We compared subjects’ performance on schema-congruent and incongruent items to assess whether the schemas indeed aided performance. At the end of the learning session, face to home allocation was superior for schema-congruent faces (*mean* = 34.65, *SD* = 1.44) than for schema-incongruent faces (*mean* = 28.21, *SD* = 6.86, *Z* = −5.74, *p* < 0.001). Subjects also retrieved the homes for schema-congruent faces faster (schema-congruent *mean* = 2965 ms, *SD* = 526 ms; schema-incongruent *mean* = 3406 ms, *SD* = 919 ms; *Z* = −4.04, *p* < 0.001) and with higher confidence (schema-congruent *mean* = 4.74, *SD* = 0.35; schema-incongruent *mean* = 4.14, *SD* = 0.56; *Z* = −5.98, *p* < 0.001) than those for schema-incongruent faces. As performance was much higher in the schema-congruent condition, this convincingly shows that subjects used the schema to their advantage.

### Face Recognition

D’ scores [z (hits) – z (false alarms); Snodgrass and Corwin (1988) and Wickens (2001)] were used to compare recognition memory sensitivity between the schema-congruent and schema-incongruent condition (see Table 1 for the raw data).

**Table 1:** **Average response frequencies for old, similar and new images, in the schema-congruent and schema-incongruent conditions**.

Item type	Response given		
	Old	Similar	New
**Schema-congruent**			
Old	16.67 (4.43)	12.48 (4.03)	6.38 (3.69)
Similar	7.71 (3.45)	16.79 (5.51)	10.98 (4.70)
New	0.79 (1.17)	4.92 (3.77)	29.75 (4.46)
**Schema-incongruent**			
Old	21.56 (5.75)	11.29 (4.23)	2.52 (2.78)
Similar	8.90 (4.97)	18.94 (5.95)	7.77 (4.51)
New	0.60 (1.05)	4.98 (3.80)	29.71 (4.11)

*Standard deviations are given in parentheses*.

Two d^′^ measures were calculated, one for global recognition (hits on old items/false alarms on new items) and one for detailed recognition (hits on old items/false alarms on lures). We performed a repeated measures ANOVA with factors MEMORY LEVEL (global, detailed) and SCHEMA (congruent, incongruent) to test the hypothesis that the use of a schema leads to shallow item encoding. As expected, a main effect of MEMORY LEVEL was found (*F*_(1,47)_ = 38.34, *p* < 0.001), showing that memory discriminability is better for global recognition (*mean* = 2.10, *SD* = 0.38) than for detailed recognition (*mean* = 0.89, *SD* = 0.38). In addition, a main effect of SCHEMA was found (*F*_(1,47)_ = 583.95, *p* < 0.001; schema-congruent *mean* = 1.31, *SD* = 0.34; schema-incongruent *mean* = 1.67, *SD* = 0.45). Follow-up tests showed that memory discriminability was much better in the incongruent condition, both for global recognition (schema-incongruent *mean* = 2.31, *SD* = 0.51; schema-congruent *mean* = 1.88, *SD* = 0.40; *Z* = −4.58, *p* < 0.001) and detailed recognition (schema-incongruent *mean* = 1.03, *SD* = 0.50; schema-congruent *mean* = 0.74, *SD* = 0.37; *Z* = −3.92, *p* < 0.001). Finally, a marginally significant interaction was found between SCHEMA and MEMORY LEVEL (*F*_(1,47)_ = 2.91, *p* = 0.095), suggesting that it might be possible that differences between the schema conditions are somewhat larger for global recognition (mean *difference* = 0.42, *SD* = 0.51) than for detailed recognition (mean *difference* = 0.30, *SD* = 0.46).

Together, these findings clearly show that memory discriminability for schema-congruent items was markedly inferior to memory for schema-incongruent items.

### Contextual Memory

To test the hypothesis that the use of a schema leads to contextually impoverished memories, we tested whether subjects could remember where the homes were located during the encoding session. Subjects were far more accurate in selecting the corresponding locations for the faces in the incongruent condition (*mean* = 19.15, *SD* = 6.33) than the congruent condition (*mean* = 9.60, *SD* = 3.87; *Z* = −5.97, *p* < 0.001). Moreover, in the incongruent condition, confidence was higher (schema-incongruent *mean*: 3.67, *SD* = 0.72 vs. schema-congruent *mean*: 2.84, *SD* = 0.69; *Z* = −5.30, *p* < 0.001) and RTs were shorter (schema-incongruent *mean*: 2472 ms, *SD* = 559 ms vs. schema-congruent *mean*: 2638 ms, *SD* = 765 ms; *Z* = −2.55; *p* = 0.011). These findings provide strong support for the idea that schema-incongruent memories were “contextually richer” as compared to the schema-congruent ones.

The questionnaire results revealed that a few subjects intentionally encoded the location of the faces during encoding, even if only for some faces. When excluding all subjects that reported the use of this feature, schema effects on accuracy and confidence were still highly significant (both *p’s* < 0.001).

The combined behavioral results thus show a clear memory disadvantage for schema-congruent items. This is apparent for recognition memory of the target items (faces) and even more so for schema-irrelevant contextual information.

### EEG Results

Memory performance suggests that retrieval of schema-incongruent items might involve more recollection (i.e., retrieval of contextual details from the learning episode) than retrieval in the schema-congruent condition. We tested this through analysis of the parietal old/new ERP effect. We first investigated whether there were differences in the average ERP amplitudes (500–800 ms time window) for the schema-congruent new and the schema-incongruent new condition. If these conditions did not differ, this would allow us to collapse these trials into a single “new” condition to which the “old” conditions could be compared. A repeated measures ANOVA with factors SCHEMA (congruent new, incongruent new) and ROI (left parietal, right parietal) revealed no main effects, nor an interaction (all *p’s* > 0.10). Therefore a single “new” condition was created.

**Figure 3:**
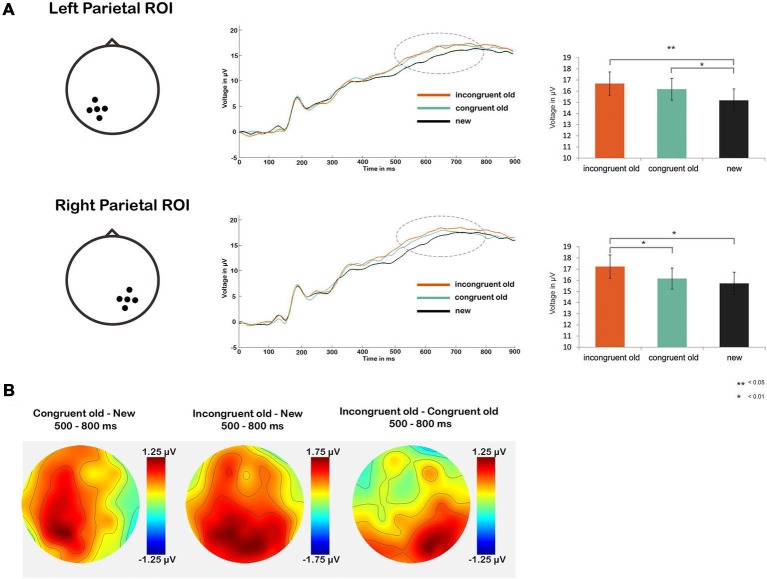
**(A)** Line graphs show average ERP waveforms for the schema-incongruent, schema-congruent and new condition, for the left (upper part) and right (lower part) parietal ROI. The dotted circles represent the time window for the parietal old/new effect (500–800 ms post-stimulus onset). Bar graphs on the right show ERP amplitudes averaged across this time window, for each condition. **(B)** Topographic maps of amplitude differences between the congruent old vs. new condition (left), incongruent old vs. new condition (middle) and incongruent old vs. congruent old condition (right).

Next, we tested our main hypothesis of a more pronounced parietal old/new effect in the schema-incongruent condition as compared to the schema-congruent condition (Figure 3). First we performed a repeated measures ANOVA with factors CONDITION (congruent old, incongruent old, new) and ROI (left parietal, right parietal). This yielded a main effect of CONDITION (*F*_(2,34)_ = 4.85, *p* = 0.011) and a CONDITION × ROI interaction (*F*_(2,34)_ = 7.03, *p* = 0.002). No main effect of ROI was found (*p* > 0.10). The significant effects were further analyzed through one-way ANOVAs with factor CONDITION (congruent old, incongruent old, new) for both ROIs separately. Significant main effects of CONDITION were found bilaterally (left ROI: *F*_(1,35)_ = 5.16, *p* = 0.008; right ROI: *F*_(1,35)_ = 4.70, *p* = 0.012). Follow up *t*-tests revealed that in the left ROI, both the schema-congruent old condition (*mean* = 16.81 μV, *SD* = 6.22 μV) and the schema-incongruent old condition (*mean* = 17.03 μV, *SD* = 6.37 μV) had a significant higher ERP amplitude than the “new” condition (*mean* = 15.56 μV, *SD* = 6.27 μV); schema-congruent vs. new: *t*_(1,35)_ = 2.23, *p* = 0.032; schema-incongruent vs. new: (*t*_(1,35)_ = 2.88, *p* = 0.007). However, the schema-incongruent and schema-congruent old conditions did not differ significantly (*p* > 0.10). Conversely, in the right ROI, the ERP amplitude in the schema-incongruent old condition (*mean* = 16.99 μV, *SD* = 6.04 μV) was higher than the amplitude in both the “new” condition (*mean* = 15.69 μV, *SD* = 5.81 μV; *t*_(1,35)_ = 2.72, *p* = 0.010) and the schema-congruent old condition (*mean* = 16.30 μV, *SD* = 5.57 μV; *t*_(1,35)_ = 2.31, *p* = 0.027). The schema-congruent old condition did not differ from the “new” condition (*p* > 0.10).

It can thus be concluded that the parietal old/new effect was more pronounced for the schema-incongruent condition than the schema-congruent condition in the right parietal region (see also the topographical map in Figure 3B, which shows that the difference between the incongruent old and congruent old conditions is, indeed, distributed around the right parietal ROI). These findings support the notion that schema-incongruent memories were contextually richer than schema-congruent memories.

### Correlations

As explained previously, the parietal old/new effect is thought to reflect the retrieval of a vivid memory trace, including contextual information from the study episode. This measure might, therefore, be expected to correlate with subjects’ context memory scores. Such a correlation would strengthen the notion that the ERP effects described in the previous section indeed reflect differences between conditions with regard to contextual memory retrieval.

**Figure 4:**
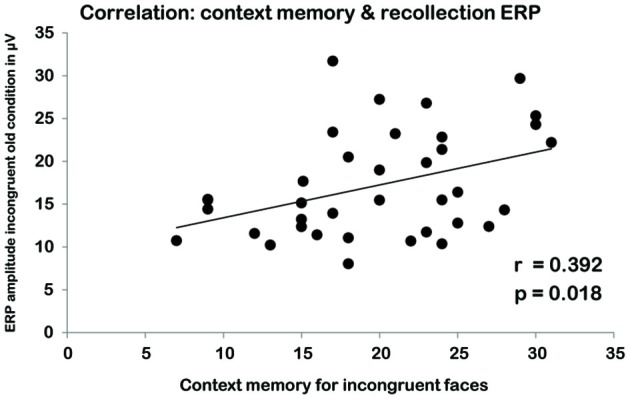
**Correlation between scores on the contextual memory task in the incongruent condition (*X*-AXIS) and EEG amplitude for the schema-incongruent old faces during the recognition task (*Y*-AXIS)**.

We correlated the average ERP amplitudes in the schema-incongruent old and schema-congruent old condition with the contextual memory scores (i.e., memory for the location of the home). A significant correlation was found in the incongruent condition where average EEG amplitude in the right ROI correlated with contextual memory scores (*r*_(34)_ = 0.392, *p* = 0.018; see Figure 4). In the left ROI, a similar, marginally significant correlation was found (*r*_(34)_ = 0.324, *p* = 0.054). The average amplitudes in the congruent old condition did not reveal any significant correlations with context memory (all *p’s* > 0.10).

## Discussion

The present study set out to test whether schemas induce shallow encoding of goal-irrelevant information. The results confirm our hypothesis: both item and context memory accuracy were strongly reduced in the schema-congruent condition. Moreover, recollection-related ERP amplitudes were larger for the schema-incongruent condition as compared to the schema-congruent condition, further supporting the hypothesis that memories for schema-congruent items are contextually impoverished.

In the present paradigm, each face stimulus was presented multiple times during the learning phase, and some level of attention to the face was always needed to select the corresponding home. These paradigm characteristics, considering also the fast processing of face stimuli by our brain (Itier and Taylor, 2004; Pegna et al., 2004), allowed, in theory, for the formation of robust memory traces for faces in both schema conditions. Still, large differences were observed between memory for the schema-congruent and schema-incongruent faces. In the face recognition test, the schema-congruent faces were less likely to be recognized, and were more often falsely endorsed as new. With regard to memory for contextual aspects of the learning episode, the difference between the schema conditions was even higher. It thus appears that the use of schemas strongly impairs memory formation, which is intriguing given the ample opportunity subjects had to form strong memory traces.

The ERP results provide further support for differences in memory quality between the schema conditions. We investigated the parietal old/new effect, which is believed to reflect recollection: the ability to retrieve source information from the study episode (Wilding, 2000). This ERP effect is generally most pronounced over parietal electrode sites and may be left lateralized (Schloerscheidt and Rugg, 1997; Wilding, 2000; Finnigan et al., 2002) or bilateral (Marzi and Viggiano, 2010; Curran and Doyle, 2011).

In the present study, the schema-congruent faces elicited a left-lateralized ERP effect, whereas the effect was bilateral in the schema-incongruent condition. Indeed, in the right parietal region the schema-incongruent ERP amplitude was significantly higher than the schema-congruent one. We like to speculate that this difference reflects the stronger recollection in the schema-incongruent condition. This notion is strengthened by the positive correlation between average ERP amplitude in the schema-incongruent old condition, especially on the right side, and the availability of spatial context memory. In fact, as spatial information processing tends to be largely lateralized to the right hemisphere (Smith et al., 1996; Corballis, 1997; Shulman et al., 2010), the right-sided ERP difference between the two schema conditions may (at least in part) reflect superior spatial memory availability in the incongruent condition.

The current findings extend previous work from our lab that showed poor memory for visual details when regularities can be extracted across the material to be learned (Sweegers and Talamini, 2014). In that study, the negative influence on storage of arbitrary (schema-irrelevant) stimulus aspects may have been related to the process of regularity extraction. We now show that such negative effects on memory encoding also occur when a pre-established schema is used. Thus, at least part of the effect is related to schema use, rather than schema formation. We, moreover, now show that such regularities, or schemas, also impair context memory retrieval, and that this is evidenced by changes in the underlying neural networks.

So how do schemas exert these negative effects on memory formation? In the present study, schemas appeared to particularly alter memory processing during the encoding phase, as poor memory for schema-items was already evident shortly after learning. This was also the case in our previous study (Sweegers and Talamini, 2014), which, in addition, showed similar retention of visual detail for schema-congruent and incongruent items during a 4 h post-encoding interval. Schema effects on retrieval processes are unlikely to have contributed importantly to the findings, as the adopted recognition task places relatively low demands on retrieval processes. Moreover, possible schema effects on response bias were accounted for by using, as our main memory measure d^′^, which is corrected for such bias. Taken together, these findings suggest negative schema effects come about during memory encoding and/or very early consolidation, probably through top-down attentional modulation.

Importantly, our findings indicate that schemas exert their influence on memory not only through facilitation of information storage, as shown previously, but also through suppression thereof. They show that such suppression occurs for schema-related information that is irrelevant to the pursuit of momentary goals. Importantly, this highlights the notion that schemas direct attention and learning in relation to momentary goals.

When such goals require memorizing novel input, schemas may help to direct attention to the relevant input and provide the background knowledge necessary for fast interpretation and storage of that input. For example, it has been shown that after extensive training on various flavor-place associations, rats can acquire a new association in a single trial (Tse et al., 2007). In another study, schema knowledge regarded the types of fabric generally used to make particular products (van Kesteren et al., 2010). Here, it was found that such a schema greatly aided in memory formation for congruent product-fabric pairs. In both the rat and the human study, memorizing the individual schema-related novel items was highly relevant in relation to task goals, suggesting that schemas help memory storage in such situations.

There are, however, also situations where schemas can sufficiently inform goal-directed behavior, so that memorizing novel input is not directly necessary. Here, schemas appear to hamper memory storage, even of material that is directly task-related. Such a mechanism might be expected to have considerable evolutionary benefit, preventing the storage of redundant information and sparing processing capacity for more important computations. Indeed, we propose the combined findings regarding schema effects on learning are best understood in terms of interactions between schemas and active goals steering attention and influencing memory processing. Thus, not only stimulus novelty (in relation to the schema), but also stimulus relevance (in relation to current goals) will influence the level of encoding. In short, depending on momentary goals, schemas may exert different influences on memory formation, steering attention so as to promote the efficient use of information processing capacity.

Interactions between schemas and momentary goals, as investigated by us, have not received much attention previously. However, several other factors influencing schema effects on memory have been identified. These factors are of considerable importance in understanding the variable effects of schemas on memory in different situations and are hence briefly discussed hereafter (see Stangor and McMillan, 1992, for a detailed account).

First and foremost, a considerably body of evidence (e.g., Pezdek et al., 1989; Neuschatz et al., 2002; Porubanova et al., 2014), amongst which a meta-analysis (Stangor and McMillan, 1992) suggests that schemas influence memories in two basic ways: on the one hand, a relation between a schema and an item will aid the retrieval thereof, but also induce a tendency to report schema-congruent items as having been seen (false memories). These effects act at the level of memory search and retrievability of the item. Accordingly, the way in which memory is cued (i.e., the retrieval paradigm) plays a role. Indeed, this effect is particularly apparent when schema information is used to cue the target memory. In view of the above, studies specifically addressing effects of schemas on memory encoding should always use measures corrected for response bias (unfortunately, this is not always the case).

Studies that do not correct for this response bias tend to find better memory for schema-congruent than schema-incongruent items. Importantly, however, studies that correct for this response bias typically find better memory for schema-incongruent than schema-congruent information, both for recall and recognition sensitivity measures, such as d^′^ (Stangor and McMillan, 1992). The benefit for schema-incongruent effects is thought to be induced by the relative novelty of the incongruent item, which favors synaptic plasticity and learning. In other words, this effect is related to effects of schemas on encoding, rather than on retrieval.

Several variables have been shown to modulate the above schema effects. One of these is the strength of the top down expectancy generated by the schema (the strength of the expectancy used to guide information processing) and the extent to which the bottom up, sensory data driven information deviates from the expectancy. The size of this relative novelty signal, or “prediction error” (Friston, 2005; Henson and Gagnepain, 2010), is thought to determine the extent of learning in neural networks, through modulation of acetylcholine (Hasselmo et al., 1996; Meeter et al., 2004). So, dependent on the size of the prediction error (and the balance with other factors modulating the schema effect) there may a relative encoding advantage for schema-incongruent compared to schema-congruent items (see van Kesteren et al., 2012 for an elaborate discussion). An important consideration in this respect is that schemas learned in the laboratory (used in most recent studies) may induce a relatively weak expectancy compared to schemas developed through real life experience, such as various social and cultural schemas (used in many older studies). Also, in many of the older studies “schema-incongruence” refers to new information being discordant with a schema-based expectation (e.g., a person in a swim suit sitting in a court of law); while in several recent studies, including our own, it refers to items bearing no relation to the schema (i.e., schema-irrelevance). As such, novelty-related schema effects might, in recent experiments, play a relatively lesser role than in some of the older literature.

At the neural level, schemas are thought to involve a network incorporating the medial prefrontal cortex (mPFC) and the hippocampus. During schema formation, the interaction between these two regions appears crucial (van Kesteren et al., 2013; Sweegers et al., 2014), biasing consolidation towards the regularities across hippocampus-dependent memories. Once schemas have been formed, further encoding of new schema-congruent information seems to particularly involve the mPFC, rather than the hippocampus (van Kesteren et al., 2013).

The neural underpinnings of schema-induced memory suppression have thus far remained undiscussed. We propose that these impairments are related to hippocampal network shifts between encoding and retrieval mode, which appear to be driven by the relative novelty of the hippocampal input and mediated by acetylcholine levels. Whereas a highly novel stimulus puts the hippocampus in encoding mode, familiar input, instead, shifts hippocampal dynamics to retrieval mode, in which little learning occurs (Hasselmo et al., 1996; Meeter et al., 2004; Wittmann et al., 2007; Schomaker and Meeter, 2012). Depending on goal-relevance, schemas may either bias attention, and therewith item processing, towards the similarities with the schema (when the schema suffices for goal achievement) or towards the unique aspects, that is, the dissimilarities to the schema (when these aspects of the stimuli are goal-relevant). In the former case, item information reaching the hippocampus would be relatively familiar, setting the hippocampus to retrieval mode. In the latter case, however, hippocampal input would contain more novel information, driving the system towards encoding mode.

A point of consideration in our study is whether the 24 h consolidation period following learning of the regularity schedule (with pairings of facial features to homes) is sufficient to allow for the build-up of a schema. Whereas various studies suggest that full hippocampo-cortical consolidation may take from several days up to several weeks (Scoville and Milner, 1957; Zola-Morgan and Squire, 1990; Bontempi et al., 1999; Talamini and Gorree, 2012), there is considerable literature showing that consolidation effects can be observed within the first 24 h post-encoding. Indeed, such effects have been observed at the level of molecular trace stabilization (Kandel, 2001), system-level recoding (Takashima et al., 2009) and behavior (Stickgold and Walker, 2007). Moreover, a previous study in our lab has shown that generalization of memorized items, which is plausibly involved in schema formation, can also be observed after an interval of a mere 4 h post-encoding (Sweegers and Talamini, 2014). We are therefore convinced that the time-window that is used in the present study allows for sufficient consolidation of the new schema to study the effects of congruency and incongruency upon new encoding.

## Conclusion

Schemas should not always be considered beneficial for learning and memory. Namely, when the storage of schema-congruent input serves no direct personal goal, schemas may in fact reduce the detailed processing of such input. Whereas the adaptive value of such a mechanism is likely related to information selection in relation to relevance for survival, an exaggerated or undue influence of schemas on information processing might counteract the flexible adaptation of already stored information. Future research should try to come to a better understanding of the circumstances under which schema use should be emphasized or rather avoided.

## Author Contributions

LMT, CCGS and GAC conceptualized and designed the study; GAC and EAMP acquired the data; GAC, EAMP and RC analyzed the data, LMT, CCGS, RC and GAC wrote the manuscript.

## Conflict of Interest Statement

The authors declare that the research was conducted in the absence of any commercial or financial relationships that could be construed as a potential conflict of interest.
